# TLR9 Negatively Regulates Intracellular Bacterial Killing by Pyroptosis in Burkholderia pseudomallei*-*Infected Mouse Macrophage Cell Line (Raw264.7)

**DOI:** 10.1128/spectrum.03488-22

**Published:** 2022-10-04

**Authors:** Matsayapan Pudla, Sucharat Sanongkiet, Peeraya Ekchariyawat, Chularat Luangjindarat, Marisa Ponpuak, Pongsak Utaisincharoen

**Affiliations:** a Department of Oral Microbiology, Faculty of Dentistry, Mahidol University, Bangkok, Thailand; b Department of Chemistry, Faculty of Science, Silpakorn University, Nakhon Pathom, Thailand; c Department of Microbiology, Faculty of Public Health, Mahidol University, Bangkok, Thailand; d Department of Microbiology, Faculty of Science, Mahidol University, Bangkok, Thailand; University of Nebraska Medical Center

**Keywords:** *Burkholderia pseudomallei*, caspase-11, macrophage defense, pyroptosis, TLR9

## Abstract

Melioidosis is a serious infectious disease caused by Burkholderia pseudomallei. This bacterium is able to survive and multiply inside the immune cells such as macrophages. It is well established that Toll-like receptors (TLRs), particularly surface TLRs such as TLR2, TLR4, and TLR5, play an essential role in defending against this bacterial infection. However, the involvement of endosomal TLRs in the infection has not been elucidated. In this study, we demonstrated that the number of intracellular bacteria is reduced in TLR9-depleted RAW264.7 cells infected with B. pseudomallei, suggesting that TLR9 is involved in intracellular bacterial killing in macrophages. As several reports have previously demonstrated that pyroptosis is essential for restricting intracellular bacterial killing, particularly in B. pseudomallei infection, we also observed an increased release of cytosolic enzyme lactate dehydrogenase (LDH) in TLR9-depleted cells infected with B. pseudomallei, suggesting TLR9 involvement in pyroptosis in this context. Consistently, the increases in caspase-11 and gasdermind D (GSDMD) activations, which are responsible for the LDH release, were also detected. Moreover, we demonstrated that the increases in pyroptosis and bacterial killing in B. pseudomallei*-*infected TLR9-depleted cells were due to the augmentation of the IFN-β, one of the key cytokines known to regulate caspase-11. Altogether, this finding showed that TLR9 suppresses macrophage killing of B. pseudomallei by regulating pyroptosis. This information provides a novel mechanism of TLR9 in the regulation of intracellular bacterial killing by macrophages, which could potentially be leveraged for therapeutic intervention.

**IMPORTANCE** Surface TLRs have been well established to play an essential role in Burkholderia pseudomallei infection. However, the role of endosomal TLRs has not been elucidated. In the present study, we demonstrated that TLR9 plays a crucial role by negatively regulating cytokine production, particularly IFN-β, a vital cytokine to control pyroptosis via caspase-11 activation. By depletion of TLR9, the percentage of pyroptosis was significantly increased, leading to suppression of intracellular survival in B. pseudomallei-infected macrophages. These findings provide a new role of TLR9 in macrophages.

## INTRODUCTION

Melioidosis is a life-threatening disease in humans and animals caused by Burkholderia pseudomallei, a Gram-negative environmental bacterium (1). The major route of infection is via exposures through broken skin, inhalation, or ingestion. Moreover, environmental conditions, such as tropical storms and specific occupations, increase the risk of exposure (2). The clinical manifestation of this infectious disease appears as abscess formation in visceral organs such as lung, liver, spleen, and soft tissues. Occasionally, infections can be acute, chronic, latent, and even progress to fatal sepsis (3). In the case of chronic infection, relapse of melioidosis patients is relatively common due to failure by the host to eradicate B. pseudomallei during the primary stages of infection, especially in the immunocompromised individuals. The overall relapse rate can range between 15% and 30% in severe melioidosis (3). One of the unique features of B. pseudomallei is its ability to invade, survive, and multiply in both phagocytic and nonphagocytic cells (4–6). After being taken into the cells, the bacteria escape from the endocytic vacuole into the cytosol and replicate. The innate immune cells, particularly macrophages equipped with several antibactericidal effects, this bacterium can avoid cellular killing via several mechanisms, including the upregulation of Toll-like receptors (TLRs) negative regulators, which leads to the suppression of inducible nitric oxide synthase (iNOS) (7–9).

The ability of immune cells, particularly macrophages, to eliminate bacteria is often associated with pattern recognition receptors (PRRs), especially TLRs. These receptors play an essential role in recognizing microbial products, followed by activating cell signaling that initiates the response against pathogens. Based on their location, TLRs can be classified into two subfamilies: cell surface TLRs (TLR1, TLR2, TLR4, TLR5, TLR6, and TLR10) and endosomal TLRs (TLR3, TLR7, TLR8, TLR9, TLR11, TLR12, and TLR13) (10). The role of several surface TLRs has been demonstrated in B. pseudomallei infection, including that of TLR2 and TLR4 *in vivo* and *in vitro* (11). Whole-blood and alveolar macrophages obtained from TLR2 and TLR4 knockout (KO) mice were less responsive to B. pseudomallei *in vitro*, suggesting that both TLRs participate in immune response against B. pseudomallei infection (12). However, decreased bacterial loads, reduced lung inflammation, and less distant-organ injury, which led to an increase of survival, were only observed in TLR2 KO mice (12). TLR5, which recognizes flagellin, was also demonstrated to be essential in innate immune response against this bacterium as the recombinant B. pseudomallei flagellin (rFliC) stimulates the production of proinflammatory cytokines (IL-1β, IL-6, and TNF-α) in healthy human whole blood (13). Furthermore, TLR5 knockout mice infected with B. pseudomallei showed significantly poorer survival than wild-type (WT) mice, suggesting that TLR5 plays a protective role in B. pseudomallei infection (14). Upon binding to TLR, the signal transduction is initiated by the recruitment of a cytoplasmic Toll/IL-1 receptor (TIR) domain-containing adaptor molecules such as myeloid differentiation primary response 88 (MyD88) and Toll/IL-1R domain-containing adaptor-inducing IFN-β (TRIF). This recruitment leads to the activation of the transcription factor NF-κB, resulting in the expression of proinflammatory cytokines and conferring the protection of the host from microbial infection (15). The MyD88-dependent pathway controls the activation of mitogen-activated protein kinases (MAPKs) and the transcription factor NF-κB, whereas the TRIF-dependent pathway mainly mediates type I IFN production (15). The involvement of TLR adaptor molecules such as MyD88 and TRIF has also been investigated in B. pseudomallei infection. MyD88-deficient mice infected with B. pseudomallei showed the increase in bacterial loads in lung, liver, and blood, suggesting that MyD88 is essential in bacterial clearance in mice (16). Unlike MyD88, the TRIF molecule is vital for regulating type I IFN-β, a key cytokine that regulates several enzymes like iNOS, thus inhibiting intracellular survival of B. pseudomallei (17). Compared with other Gram-negative bacteria, B. pseudomallei fails to stimulate IFN-β in Raw264.7 cells, resulting in suppression of the iNOS expression. Several lines of evidence suggest that B. pseudomallei induces less cytokine production and NF-κB activation in both phagocytic and nonphagocytic cell lines (17–19). Among the virulence factors of the B. pseudomallei, TssM, was demonstrated to interfere with IκBα ubiquitination in the infected cells, leading to the attenuation of NF-κB and IFN-β activation (20). One of the mechanisms by which this bacterium was able to suppress IFN-β was also demonstrated. B. pseudomallei can interfere with the TRIF signaling/IFN-β production pathway by upregulating several negative regulators of TRIF signaling molecule such as SARM and SIRP-α, which leads to the inhibition of IFN-β, resulting in an increased intracellular replication of the bacteria (8, 9). Recent evidence demonstrated that TLR4/TRIF-mediated type-I-IFN production is also essential for regulating caspase-11 (21–23). This enzyme can sense LPS of Gram-negative bacteria in the cytosol and forming complex consisting of inactive procaspase-11 and LPS, leading to the activation of caspase-11 (24). Once caspase-11 is activated, it cleaves gasdermin D (GSDMD) and initiates pyroptosis by forming pores on the cell membrane, causing swelling and lysis, followed by the release of cytoplasmic contents such as the inflammatory cytokine interleukin-1β (IL-1β) (25–27). Pyroptosis, in the caspase-11 dependent manner, was also observed in mouse lung epithelial cells infected with Burkholderia thailandensis both *in vivo* and *in vitro* (28). Several reports also showed that caspase-11 can protect the mice from lethal challenges with B. thailandensis and B. pseudomallei (29, 30). The intracellular replication of Legionella pneumophila was also increased in the caspase-11-deficient bone-marrow-derived macrophages (BMDMs), suggesting that caspase-11 can restrict intracellular replication of the bacteria (31). The mechanisms by which caspase-11 inhibits the intracellular growth of bacteria have not been fully elucidated. Another possible mechanism might be the presence of GSDMD. Recent evidence shows that GSDMD also exhibits bactericidal activity by directly binding to several bacteria, including B. thailandensis (32, 33).

Surface TLRs have been well established to participate in pyroptosis. However, very little information on the interrelationship of endosomal TLRs, mainly TLR9, and this form of cell death in bacterial infection has been established. TLR9 is known to recognize bacterial DNA and plays a role in an innate immune response against bacterial infection. Zhan et al. demonstrate that *TLR9*^−/−^ mice exhibited shortened survival, an increased cytokine storm, and more severe Salmonella *hepatitis* than the WT mice, suggesting that TLR9 may act as a negative regulator suppressing cytokine production (34). Moreover, TLR9-deficient mice have also been shown to reduce the clearance of Streptococcus pneumoniae from the lungs, implying that TLR9 may also involve in bacterial clearance (35). However, the interrelation of TLR9 and pyroptosis has not been elucidated. In this study, our results presented here show that TLR9 negatively regulates pyroptosis via caspase-11 activation in Raw264.7 cells upon B. pseudomallei infection.

## RESULTS

### TLR9, but not TLR3 and TLR7, participates in B. pseudomallei-infected Raw264.7 macrophage killing.

**FIG 1:**
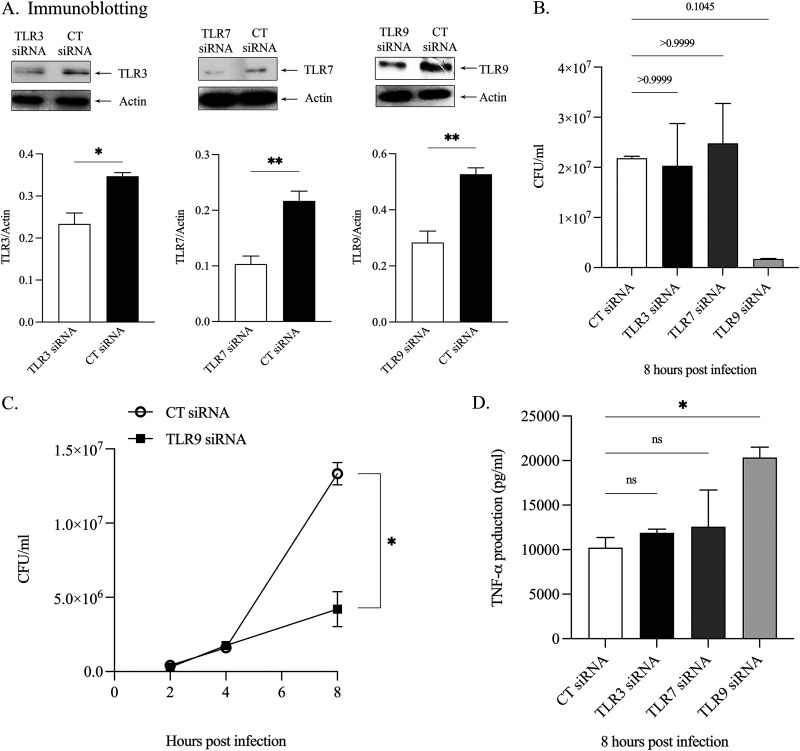
Depletion of TLR9 enhances bacterial killing and increases TNF-α production in B. pseudomallei infection. Raw264.7 macrophages were transfected with siRNAs against TLR3, TLR7, and TLR9 or control siRNAs (CT siRNA). At 48 h posttransfection, the cells were infected with B. pseudomallei at MOI of 2. (A) Representative images of protein expression by immunoblotting were shown. The intensities of these endosomal TLRs and Actin were quantified using ImageJ. Data are representative of those from three independent experiments. The Student's *t* test was used to compare the protein expression. *, *P* < 0.05 and **, *P* < 0.01. (B) At 8 h postinfection, the infected cells were lyzed and the number of CFU was determined by a standard antibiotic protection assay. Data are mean ± SEM from three independent experiments. All relative CT siRNA were analyzed using a Kruskal-Wallis followed by a Dunn’s multiple-comparison test. The differences between the CT siRNA and the experimental treatments (TLR3, TLR7, and TLR9 siRNAs) were not significant as shown in *P* values. (C) The macrophages were transfected with siRNAs against TLR9 or control siRNAs (CT siRNA) prior to infection with B. pseudomallei at MOI of 2. At the indicated times after infection, the infected cells were lyzed and the number of CFU was determined by a standard antibiotic protection assay. Data are mean ± SEM from three independent experiments. The Student's *t* test was used to compare CFU data. *, *P* < 0.05. (D) Detection of TNF-α production from the supernatant of infected cells was determined by ELISA 8 h postinfection. Data are mean ± SEM from three independent experiments. All relative to CT siRNA was analyzed using an ordinary one-way ANOVA followed by a Tukey’s multiple-comparison test. *, *P* < 0.05. CT siRNA, control siRNA.

Given that it is known that the surface TLRs participate in immune response against B. pseudomallei infection (12), the involvement of endosomal TLRs (TLR3, TLR7, and TLR9) were further investigated in B. pseudomallei-infected RAW264.7 cells. The endosomal TLRs were depleted by siRNAs. To test the silencing efficiency, the protein expression of TLR3, TLR7, and TLR9 was analyzed by immunoblotting, and partial depletion was confirmed in all the three TLRs (Fig. 1A). Interestingly, the trend of intracellular bacterial burden subversion was observed only in B. pseudomallei-infected TLR9-depleted Raw264.7 cells compared with those of control siRNA-transfected cells (Fig. 1B). These data suggested that TLR9 facilitates the dissemination of B. pseudomallei-infected Raw264.7 cells. To verify the kinetics of the intracellular bacteria replication, the number of intracellular bacteria at different time intervals in TLR9-depleted Raw264.7 cells was analyzed. As shown in Fig. 1C, at 2 h and 4 h postinfection, the numbers of intracellular bacteria were not different between B. pseudomallei-infected TLR9-depleted Raw264.7 cells and the control siRNA-transfected cells, but they were significantly different after 8 h. This result implies that TLR9 does not interfere with the early bacterial internalization but inhibits the suppression of intracellular bacteria at a late time point due to the intracellular killing of the macrophages. Therefore, the expression of TLR9 causes an impairing of the intracellular killing of B. pseudomallei infection.

To further evaluate the role of endosomal TLRs in inflammatory response after B. pseudomallei infection, we determined the level of TNF-α in the supernatant of the infected cells. As shown in Fig. 1D, the secretion of TNF-α was also significantly higher at 8 h postinfection only in the depletion of TLR9, suggesting the expression of this TLR also involves in the inhibition of proinflammatory cytokine production.

### Inhibition of TLR9 expression promotes the intracellular bacterial killing by enhancing pyroptosis.

**FIG 2:**
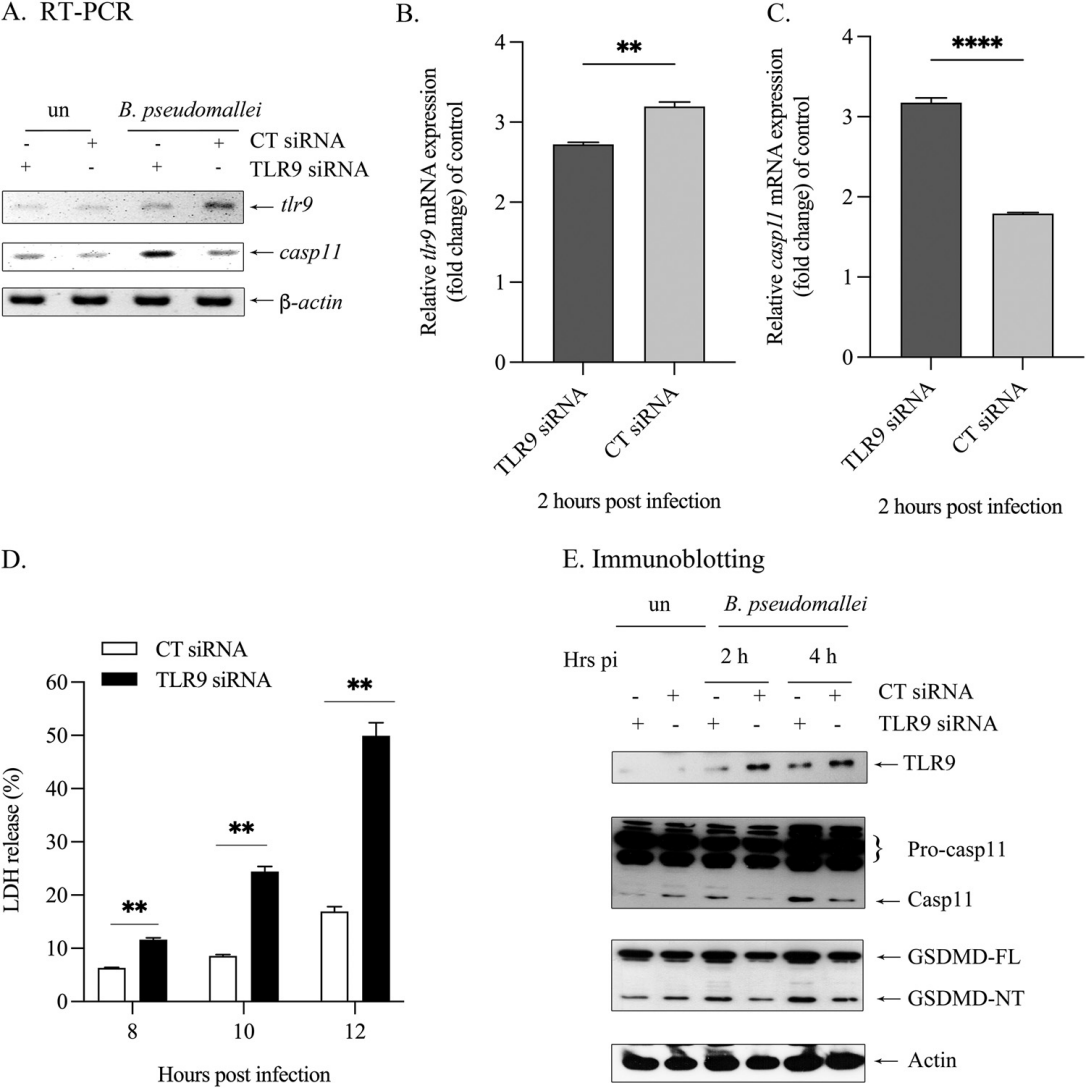
The increase in caspase-11 and GSDMD-NT activation and pyroptosis in TLR9-depleted Raw264.7 macrophages infected with B. pseudomallei. (A to C) Gene expression of *tlr9* and *casp11* in B. pseudomallei-infected TLR9-depleted Raw264.7 macrophages. Representative images of gene expression by RT-PCR were shown at 2 h postinfection. The intensities of *tlr9* and *casp11* mRNA expression were quantified using ImageJ. The graphs showed the relative mRNA expression (fold change) of control. Data are mean ± SEM from three independent experiments. The Student's *t* test was used to compare the relative mRNA expression. **, *P* < 0.01 and ****, *P* < 0.0001. (D) At the indicated times after infection, the supernatant of infected cells was subjected to LDH assay. LDH release is expressed as the percent cytotoxicity of pyroptosis. Data are mean ± SEM from three independent experiments. The Student's *t* test was used to compare LDH release. **, *P* < 0.01. (E) The protein expression of B. pseudomallei-infected TLR9-depleted Raw264.7 macrophages was determined by immunoblotting. Data are representative of those from three independent experiments. un, uninfected cells.

In murine macrophages, noncanonical inflammasome (caspase-11) plays a protective role during lung infection with B. thailandensis and B. pseudomallei via pyroptosis (28–30). To further examine the expression of caspase-11 in the depletion of TLR9, first, TLR9 partial depletion by siRNA was confirmed by RT-PCR and immunoblotting (Fig. 2A, B, and E). In the TLR9-depleted RAW264.7 cells infected with B. pseudomallei, we observed increases in caspase-11 gene expression as well as caspase-11 activation (Fig. 2A, C, and E). Moreover, to determine if an increase in intracellular killing in TLR9-deficient Raw264.7 cells is due to pyroptosis, the release of lactate dehydrogenase (LDH) was measured in B. pseudomallei-infected TLR9-depleted Raw264.7 cells. As shown in Fig. 2D, the release of LDH from silencing of TLR9 was gradually increased in a time-dependent manner and the level of LDH was significantly higher compared to those of control siRNA-transfected cells. Gasdermin D is the key substrate for caspase-11 and can be cleaved by caspase-11 to create an N-terminal fragment that functions as a key determinant for proinflammatory cell death known as pyroptosis (36). As expected, upregulation of caspase-11 was associated with a higher level of GSDMD cleavage (GSDMD-NT) in macrophages deficient in TLR9 compared with those of control siRNA-transfected cells (Fig. 2E). Together, these results indicated that TLR9 facilitates B. pseudomallei to evade macrophage killing by suppression of caspase-11, which results in the inhibition of pyroptosis.

### B. pseudomallei induces the expression of IFN-β in TLR9 depletion.

**FIG 3:**
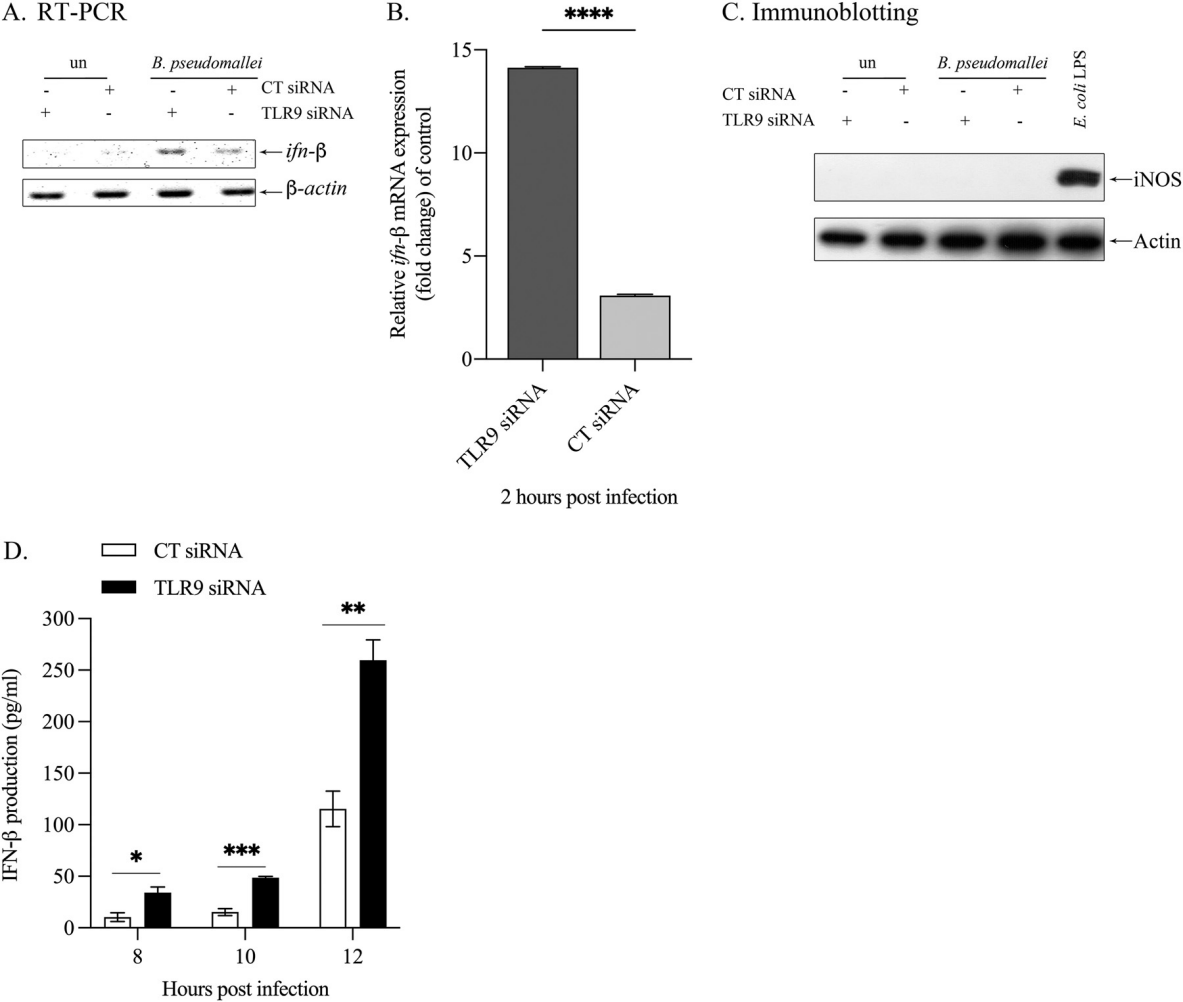
Enhancement of IFN-β gene expression and secretion in TLR9-depleted Raw264.7 macrophages infected with B. pseudomallei. (A) Representative image of gene expression was shown at 2 h postinfection. (B) The intensity of *ifn-β* mRNA expression was quantified using ImageJ. The graph showed the relative mRNA expression (fold change) of control. Data are mean ± SEM from three independent experiments. The Student's *t* test was used to compare the relative mRNA expression. ****, *P* < 0.0001. (C) At 8 h postinfection, the iNOS protein expression was determined by immunoblotting. Escherichia coli LPS was used as a positive control. Data are representative of those from three independent experiments. (D) The level of IFN-β production was determined in the supernatant of infected cells by ELISA. Data are mean ± SEM from three independent experiments. The Student's *t* test was used to compare the level of IFN-β production. *, *P* < 0.05; **, *P* < 0.01; and ***, *P* < 0.001. un, uninfected cells.

Previously, our group reported that B. pseudomallei*-*infected Raw264.7 cells fails to activate the IFN-β production, thus leading to the existence and multiplication of this intracellular pathogen inside the cells (17). This cytokine is also known to play an essential role in regulation of caspase-11 (21, 22). To elucidate whether TLR9 negatively regulates IFN-β production in B. pseudomallei infection, the mRNA expression and the level of IFN-β secretion were determined by RT-PCR and ELISA, respectively. The results showed that both mRNA expression (Fig. 3A and B) and the level of IFN-β production (Fig. 3D) were significantly increased in TLR9-depleted cells, suggesting that TLR9 plays a crucial role in the negative regulation of IFN-β in B. pseudomallei infection. In contrast, the upregulation of IFN-β production was not observed in the depletion of TLR3 and TLR7 (data not shown). Although an increase of IFN-β was observed in TLR9-depleted cells, IFN-β at this level did not cause the increased expression of iNOS in this context, as shown in Fig. 3C. Therefore, the increase in B. pseudomallei intracellular killing in TLR9-depleted cells was more likely due to an induction of pyroptosis.

### A neutralizing antibody against IFN-β attenuates the ability of macrophage killing via pyroptosis in TLR9-depleted Raw264.7 cells.

**FIG 4:**
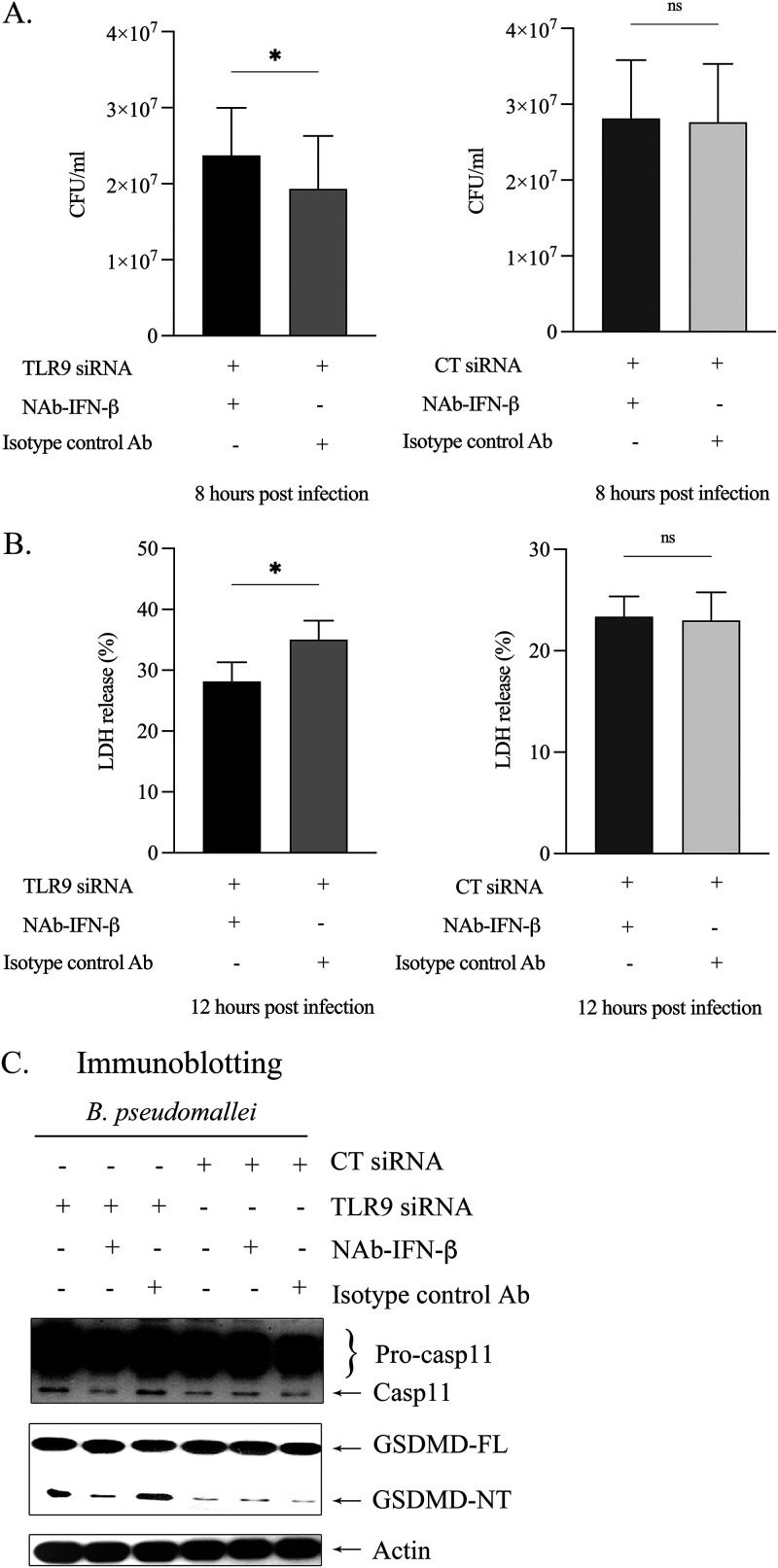
A neutralizing antibody against IFN-β increases B. pseudomallei intracellular survival by suppressing pyroptosis in TLR9-depleted Raw264.7 macrophages. TLR9-depleted cells were pretreated with 10 μg/mL of NAb-IFN-β Biolegend) or isotype control antibody (Biolegend) for 15 min before infection with B. pseudomallei at MOI of 2. (A) At 8 h postinfection, the infected cells were lyzed and the number of CFU was determined by a standard antibiotic protection assay. Data are mean ± SEM from three independent experiments. The Student's *t* test was used to compare CFU data. *, *P* < 0.05. (B) At 12 h postinfection, the supernatant of infected cells was subjected to LDH assay. LDH release is expressed as the percent cytotoxicity of pyroptosis. Data are mean ± SEM from three independent experiments. The Student's *t* test was used to compare LDH release. *, *P* < 0.05. (C) At 4 h postinfection, the protein expression was determined by immunoblotting. Data are representative of those from three independent experiments. CT siRNA, control siRNA.

To investigate whether the increase in IFN-β secretion is involved in pyroptosis, the B. pseudomallei-infected TLR9-depleted Raw264.7 cells were pretreated with a neutralizing antibody against IFN-β. The number of intracellular bacteria, as well as LDH released, were analyzed. As shown in Fig. 4A, the number of intracellular bacteria survived was significantly increased in the presence of a neutralizing antibody against IFN-β (NAb-IFN-β). This result was also correlated with a decrease in the percentage of LDH release (Fig. 4B). Consistently, NAb-IFN-β was also able to attenuate the activation of caspase-11 and GSDMD compared with that of the isotype control antibody (Fig. 4C), suggesting that the increase in IFN-β production observed in TLR9-depleted Raw264.7 cells can mediate the activation of caspase-11 and GSDMD, leading to pyroptosis and the elimination of intracellular B. pseudomallei in macrophages.

## DISCUSSION

In contrast to surface TLRs, nucleic acid-sensing TLRs, which include TLR 3, TLR7, TLR8, TLR9, and TLR13 localize within the endosomal compartments of immune cells and recognize double-stranded RNA (dsRNA), single-stranded RNA (ssRNA), and DNA derived from viruses, bacteria, fungi, and parasites, respectively (37). Endosomal TLRs can only function in acidified endosomes containing various proteases (10, 37, 38). Upon recognizing foreign nucleic acids, these receptors initiate innate immune responses to ensure host protection by activation of proinflammatory cytokines production. A recent report showed that chloroquine diphosphate, which can alkalize acidic conditions inside the cells, could also suppress intracellular replication of B. pseudomallei (39). This result may imply that endosomal TLRs might play a significant role in B. pseudomallei*-*infected cells. In our study, inhibition of intracellular B. pseudomallei (Fig. 1B and C) and increase of proinflammatory cytokines production such as TNF-α and IFN-β (Fig. 1D; Fig. 3D) were observed only when TLR9 but not TLR3 or TLR7 was depleted, suggesting that TLR9 participates in intracellular killing of B. pseudomallei in Raw264.7 cells. The increased ability to kill bacteria and rapidly increase proinflammatory cytokines production such as TNF-α was also observed in TLR9^−/−^ mice infected with Pseudomonas aeruginosa (40). These results suggested that TLR9 may negatively regulate cytokine production and suppress intracellular bacterial killing. In contrast, TLR9^−/−^ mice infected with Salmonella enterica serovar Typhimurium or Legionella pneumophila promoted bacterial loads in liver and lung, respectively, indicating TLR9 is involved in bacterial clearance of these two bacteria (34, 41). Therefore, the effect of TLR9 may be distinct between each type of bacteria. Several mechanisms can restrict the intracellular growth of bacteria in immune cells. Among them, noncanonical caspase-11 has been shown to play an essential role in pyroptosis. This process is directly related to the inhibition of intracellular replication and bacterial clearance in many Gram-negative bacterial infections. For example, macrophages from caspase-11-deficient mice showed a higher number of intracellular L. pneumophila than macrophages from WT mice, suggesting the role of caspase-11 in the inhibition of intracellular replication of this bacterium (31). Another report also showed that the increase in bacterial burden in several organs, such as spleen and liver, is also observed in caspase-11-deficient mice infected with B. thailandensis (28).

In our study, we further demonstrated that TLR9 could negatively regulate pyroptosis, as shown by the increased level of LDH release in B. pseudomallei*-*infected TLR9-depleted Raw264.7 cells (Fig. 2D). The induction of LDH release was also directly correlated with the upregulation of caspase-11 mRNA expression and caspase-11 activation (Fig. 2A, C, and E). The activation of this enzyme is initiated by forming the complex with LPS of Gram-negative bacteria, resulting in the cleavage of GSDMD to generate a 32 kDa N-terminal fragment (GSDMD-NT), which induces pyroptosis by being assembled into pores, resulting in the loss of ionic gradient, cell membrane rupture, and pyroptosis (26, 27). Furthermore, GSDMD can directly lyze the extracellular bacteria such as Escherichia coli or Staphylococcus aureus by binding to cardiolipin (a lipid found in bacterial cell membrane) and oligomerize to form pore on the bacterial cell membrane (32, 42, 43). Recent evidence also demonstrated that the cleavage of recombinant GSDMD to GSDMD-NT by caspase-1 could directly kill B. thailandensis (33). Our study also showed that the level of GSDMD-NT was higher in TLR9-depleted Raw264.7 cells infected with B. pseudomallei (Fig. 2E). Therefore, the suppression of intracellular bacteria replication in TLR9-depleted Raw264.7 cells may be due to the increased level of active GSDMD. Together, our results suggested that B. pseudomallei prevents pyroptosis via TLR9, which can then avoid intracellular killing by macrophages.

Expression of procaspase-11 is regulated, at least in part, by type I IFN (21). It was shown that *Ifnar1* deficiency completely abolishes procaspase-11expression in response to LPS, IFN-β, or EHEC infection, suggesting that IFN-β can regulate procaspase-11 expression (22). Compared with other Gram-negative bacteria, we demonstrated that B. pseudomallei failed to activate IFN-β production in Raw264.7 cells (17). However, the level of IFN-β production was significantly augmented in TLR9-depleted Raw264.7 cells, suggesting that TLR9 negatively regulates IFN-β production (Fig. 3). The increase in IFN-β production is also directly correlated with the increase of caspase-11 mRNA expression. Several reports showed that the production of type-I-IFN is mediated via the TRIF pathway, which is responsible for caspase-11 activation (21, 22). However, some reports indicated that treatment of IFN-β alone could only upregulate caspase-11 expression, but activating this enzyme also requires signaling from TLRs (21). In our study, we demonstrated that, in the presence of a neutralizing antibody against IFN-β, the level of caspase-11 and GSDMD activation (Fig. 4C) was attenuated, which also correlated with the suppression of pyroptosis (Fig. 4B) and increase in the number of intracellular of B. pseudomallei (Fig. 4A) as observed in TLR9-depleted RAW264.7 cells. These results implied the role of IFN-β production in the context of B. pseudomallei infection in TLR9-depleted cells in pyroptosis.

B. pseudomallei is an intracellular bacterium that can avoid killing inside the macrophages. Previous reports from our group showed that this bacterium could inhibit IFN-β production by upregulating several negative regulators of the TRIF pathway such as SARM, SIRP-α, leading to inhibiting intracellular killing in the macrophages (8, 9). In this study, we further demonstrate the significant role of TLR9 in B. pseudomallei*-*infected Raw264.7 cells. Signaling from this endosomal TLR can inhibit IFN-β production, leading to suppression of caspase-11 activation, which can facilitate this bacterium to survive inside the macrophages. It is possible that TLR9 could upregulate negative regulators of TLRs signaling, which lead to inhibition of proinflammatory cytokines production. A recent report supported our hypothesis, which showed that TLR9 could upregulate the expression of A20 (tumor necrosis factor-alpha [TNF-a]-induced protein 3 [TNFAIP3]), an inhibitor of the NF-κB pathway. Moreover, A20 also acts as a negative regulator of TLR signaling, resulting in the inhibition of proinflammatory cytokine (44). Therefore, the negative regulators of the TLRs signaling pathway regulated via TLR9 that influence the control of B. pseudomallei replication in Raw264.7 cells are under investigation in our laboratory.

## MATERIALS AND METHODS

### Cell line and culture condition.

Mouse macrophage cell line (Raw264.7) (ATCC) was cultured in advanced Dulbecco's modified Eagle's medium (DMEM) (HyClone) supplemented with 10% fetal bovine serum (HyClone) and 1% l-glutamine (Gibco Labs) at 37°C under a 5% CO_2_ atmosphere.

### Bacterial strain.

B. pseudomallei (1026b strain) was cultured in Luria-Bertani (LB) broth at 37°C with agitation at 150 rpm (45). The log-phase bacterial cultures were washed twice with phosphate-buffered saline (PBS) and adjusted to the desired concentration by measuring the optical density at 650 nm. The number of CFU was calculated from the precalibrated standard curve.

### Transfection of Raw264.7 cells with small interfering RNA.

siRNA treatment was performed with siRNA (Invitrogen) against mouse TLR3: CCUGAUGAUCUUCCCUCUAACAUAA; UUAUGUUAGAGGGAAGAUCAUCAGG; TLR7: GCUGCAGGUCAUCCAUGCAUCUAUA; UAUAGAUGCAUGGUAGACCUGCAGC; TLR9: CCAACAUCCUGGUUCUAGAUGCUAA; UUAGCAUCUAGAACCAGGAUGUUGG or control siRNA (Qiagen). siRNAs (1.5 μg) were nucleofected into Raw264.7 cells with 90 μL of Nucleofector solution kit V (Amaxa, London, UK) (46). After transfection, the cells were transferred into a new flask containing 10 mL complete media and incubated at 37°C followed by a medium change at 6 h after incubation. The cells were then used in assays after 48 h of transfection with siRNAs.

### Infection of Raw264.7 cells.

An overnight culture of the cells (5 × 10^5^ cells/well) in a 6-well plate was infected with bacteria at a multiplicity of infection (MOI) of 2 for 1 h. The cells were washed twice with 1 mL of PBS to remove extracellular bacteria, and residual bacteria were killed by incubating the cells in DMEM containing 250 μg/mL kanamycin (Gibco) for 2 h. Thereafter, the infection was allowed to continue in the medium containing 20 μg/mL of kanamycin until the experiment was terminated (6).

### Quantification of intracellular bacteria.

Intracellular bacterial replication was determined using a standard antibiotic protection assay (6). In brief, at the time indicated in the figure, the infected cells were washed with PBS, and intracellular bacteria were liberated by lyzing the cells with 0.1% Triton X-100. The number of released bacteria was determined by CFU/mL.

### Reverse transcription-PCR.

According to the manufacturer's instruction, RNA was isolated from the infected cells (GE Healthcare). The cDNAs were further converted using the avian myeloblastosis virus (AMV) reverse transcription enzyme (Promega). The primer sequences were: for *tlr9*; (F) GCACAGGAGCGGTGAAGGT (R) GCAGGGGTGCTCAGTGGAG, *ifn-β*; (F) TCCAAGAAAGGACGAACATTCG (R) TGAGGACATCTCCCACGTCAA, *caspase-11*; (F) CAGTGACAAGCGTTGGGTTT (R) ACTCCATGCCCTCTGCTGTA, *β-actin*; (F) CCAGAGCAAGAGAGGTATCC (R) CTGTGGTGGTGAAGCTGTAG. The amplified products were then electrophoresed using 1.5% agarose gel. The agarose gel was stained with ethidium bromide and visualized under a UV lamp.

### Immunoblotting.

The infected cells were lyzed in lysis buffer containing 20 mM Tris, 100 mM NaCl, and 1% NP40. The lysates were separated on 7%, 10%, and 15% SDS-PAGE gels. Proteins were transferred onto a nitrocellulose membrane (Amersham Biosciences). The nonspecific binding sites on the membrane were blocked with 5% blocking solution (Roche Diagnostics) for 1 h before proteins were allowed to react with specific primary antibodies against TLR3 (Thermo Fisher Scientific), TLR7 (R&D Systems), iNOS (Santa Cruz Biotechnology), TLR9 (Santa Cruz Biotechnology), caspase-11 (Abcam), GSDMD (Abcam), and Actin (Merck Millipore) at 4°C overnight. The membranes were washed three times with 0.1% PBS with Tween 20 (PBST) and incubated with horseradish peroxidase-conjugated goat anti-rabbit IgG or goat anti-mouse IgG (R&D Systems) for 1 h at room temperature. Thereafter, the membranes were washed four times with 0.1% PBST before a chemiluminescence substrate (Roche Diagnostics) was added and protein brands were detected by enhanced chemiluminescence.

### LDH assay.

The release of LDH in the culture supernatants was measured by cytotoxicity assay to determine the level of pyroptosis. At the indicate time intervals, the supernatants of infected cells were collected and LDH activity was detected by using the CytoTox 96 nonradioactive cytotoxicity assay (Promega) according to the manufacturer’s instructions. The supernatants were applied to each well of flat-bottom 96-well enzymatic assay plate (NUNC Brand Product). The 50 μL of reconstituted substrate mix was added and incubated at room temperature in the dark for 20 min. To stop the reaction, 50 μL of stop solution was applied to each well. The LDH measurement are recorded absorbance at 490 nm. For maximum LDH release, uninfected cells were added to 10× lysis solution and the LDH detection was performed in same manner. The percentage of LDH release was calculated followed by the formula: % LDH release = (experimental LDH release – spontaneous LDH release/maximum LDH release) ×100.

### Determination of IFN-β and TNF-α production.

According to the manufacturer's instruction, the cytokine levels in the supernatant of infected Raw264.7 macrophages were measured by TNF-α (e-Bioscience) and IFN-β (R&D Systems) ELISA kits.

### Statistical analysis.

All experiments were performed at least three independent times. The results were expressed as mean ± SEM. All data were analyzed by the Prism software (GraphPad) by using Student's *t* test or one-way ANOVA test followed by a *post hoc* multiple-comparison test based on specific experiments. Asterisks indicate statistically significant differences based on *P*-values as indicated here: * for *P* < 0.05; ** for *P* < 0.01; *** for *P* < 0.001; and **** for *P* < 0.0001.
